# Comparison of the diagnostic accuracy of resistin and CRP levels for sepsis in neonates and children: a systematic review and meta-analysis

**DOI:** 10.3389/fped.2025.1555671

**Published:** 2025-05-09

**Authors:** Fen Xu, Jun Luo, Wenbin Li

**Affiliations:** ^1^ Department of Neonatology, Shenzhen Baoan Women's and Children's Hospital, Guangdong, China; ^2^ Department of Pediatrics, Tongji Hospital, Tongji Medical College, Huazhong University of Science and Technology, Wuhan, China

**Keywords:** resistin, CRP, paediatric sepsis, neonatal sepsis, meta-analysis

## Abstract

**Background:**

Resistin (RETN) levels are potential diagnostic markers for sepsis in neonates and children. However, studies have yielded inconsistent results. This study aimed to compare the diagnostic accuracy of RETN levels with that of C-reactive protein (CRP) levels in the diagnosis of paediatric and neonatal sepsis through a comprehensive review of recent literature.

**Methods:**

A standard methodology for systematic reviews and meta-analyses was followed. The PubMed, Embase and Cochrane databases were searched from January 1996 to October 2024 (PROSPERO CRD42024621872). Eligible studies were selected and analysed using Review Manager 5.4 and STATA 17. Meta-DiSc version 1.4 was used to describe and calculate the sensitivity, specificity, summary receiver operating characteristic (SROC) curves and areas under the curves (AUCs). SROC curve analysis was used to summarize the overall performance.

**Results:**

A total of 437 neonates and children were included in six identified studies, all of which demonstrated reasonable methodological quality. The pooled sensitivity for the RETN level was 0.88 [95% confidence interval (CI), 0.83–0.92], which surpassed that of the CRP level at 0.85 (95% CI, 0.79–0.90). However, the pooled specificity for the RETN level was 0.78 (95% CI, 0.71–0.83), which was lower than that of the CRP level at 0.84 (95% CI, 0.77–0.90). Furthermore, the SROC curves for RETN and CRP in predicting sepsis in neonates and children indicated high predictive abilities, with AUC values of 0.925 and 0.945, respectively.

**Conclusions:**

The current evidence suggests that the RETN level is a valuable biomarker for detecting paediatric and neonatal sepsis.

**Systematic Review Registration:**

https://www.crd.york.ac.uk/PROSPERO/, identifier [CRD42024621872].

## 1 Introduction

Despite recent advancements in neonatal intensive care units (NICUs) and paediatric intensive care units (PICUs), sepsis continues to be a significant contributor to morbidity and mortality, particularly in preterm infants (1, 2). More than half of fatalities in children under five years of age are attributed to infectious diseases, such as pneumonia and diarrhoea, which can precipitate sepsis (3). The prevalence of sepsis-related morbidity and mortality in children ranges from 6.2% to 23.1% and from 9% to 20.0%, respectively (4). Clinicians are actively seeking clinical indicators or biomarkers to facilitate early diagnosis and treatment of paediatric and neonatal sepsis. However, early diagnosis remains challenging because of the nonspecific clinical signs and symptoms of sepsis in neonates and children. Therefore, timely and precise diagnosis of sepsis is crucial for implementing appropriate antibiotic therapy to mitigate the risk of adverse outcomes.

Resistin (RETN) was first identified and named for its involvement in insulin resistance in 2001 (5). In humans, RETN appears to be predominantly secreted by macrophages rather than adipocytes (6, 7). The proinflammatory adipokine RETN has subsequently been found to be elevated during sepsis in an intensive care unit (ICU) (8). RETN enhances inflammatory responses by activating nuclear factor kappa-B (NF-*κ*B) signaling through toll-like receptor 4 (TLR4), triggering interleukin-6 (IL-6) and tumor necrosis factor-alpha (TNF-α) production (9). C-reactive protein (CRP), a classic acute-phase protein, increases from ∼1 μg/ml to potentially 1,000-fold higher levels during inflammation. This rapid response begins within 6–8 h, peaks at 24–48 h, and makes CRP valuable for clinical monitoring (10–12). Khttab et al. reported that RETN was a valuable biomarker for diagnosing neonatal sepsis, and its levels were correlated with indicators of disease severity. At a cut-off level of 22.8 ng/ml, RETN demonstrated a sensitivity of 98.3% and a specificity of 99.97% (13). However, Aliefendioglu et al. reported moderate diagnostic performance for RETN, with a sensitivity of approximately 73.7% and a specificity of approximately 45.8%, yielding positive and negative predictive values of 68.3% and 52.4%, respectively. The findings also indicated that the diagnostic utility of RETN was limited compared with that of other inflammatory markers, including CRP, procalcitonin, and interleukin-6 (IL-6) (14, 15). In view of this controversy, a more thorough review encompassing the latest literature is warranted to compare the diagnostic accuracy of RETN levels with that of CRP levels in diagnosing paediatric and neonatal sepsis.

Consequently, we conducted a systematic review and meta-analysis to examine the correlation between elevated RETN levels and the risk of sepsis in neonates and children. Our primary objective was to systematically and quantitatively assess all published studies regarding the diagnostic application of RETN and CRP levels for sepsis in these populations.

## 2 Methods

### 2.1 Retrieval and selection of studies

The common approach to a computer-aided literature search was used to search PubMed, EMBASE (http://www.embase.com/) and the Cochrane Library (http://www.the-cochranelibrary.com/view/0/index.html) for relevant citations from January 1996 to October 2024. The search strings are included in the Supplementary Material. We also examined the references of known articles. A prospective registration was made in PROSPERO (ID CRD42024621872; available at https://www.crd.york.ac.uk/PROSPERO/).

The following criteria were used to select studies for inclusion in our meta-analysis: (1) observational or interventional studies; (2) studies measuring RETN and/or CRP levels; (3) neonatal and paediatric patients with sepsis were classified as the experimental group, while participants suspected of having sepsis but not confirmed were classified into the control group; (4) sufficient data to calculate the outcome metrics (true positive [TP], false positive [FP], true negative [TN], and false negative [FN]); (5) blood measurements of RETN must have been conducted at the time of clinical presentation with suspected sepsis, prior to the initiation of antimicrobial therapy, or in asymptomatic neonates or children at the time of the inclusion in the study; and (6) sepsis must have been defined as the outcome. Neonatal sepsis was defined as a positive microbial blood culture in the studies reviewed. Paediatric sepsis was defined on the basis of the criteria established by the American College of Chest Physicians/Society of Critical Care Medicine. The exclusion criteria included the following: (1) abstracts, reviews, and animal studies; (2) diagnostic methods for sepsis did not involve the measurement of RETN levels; (3) inadequate data to derive outcome metrics (TP, FP, TN, and FN); (4) bioinformatics analyses and duplicate publications; and (5) studies published in a language other than English. Article selection was conducted independently by two investigators to ensure a high level of accuracy.

### 2.2 Data extraction

**Figure 1:**
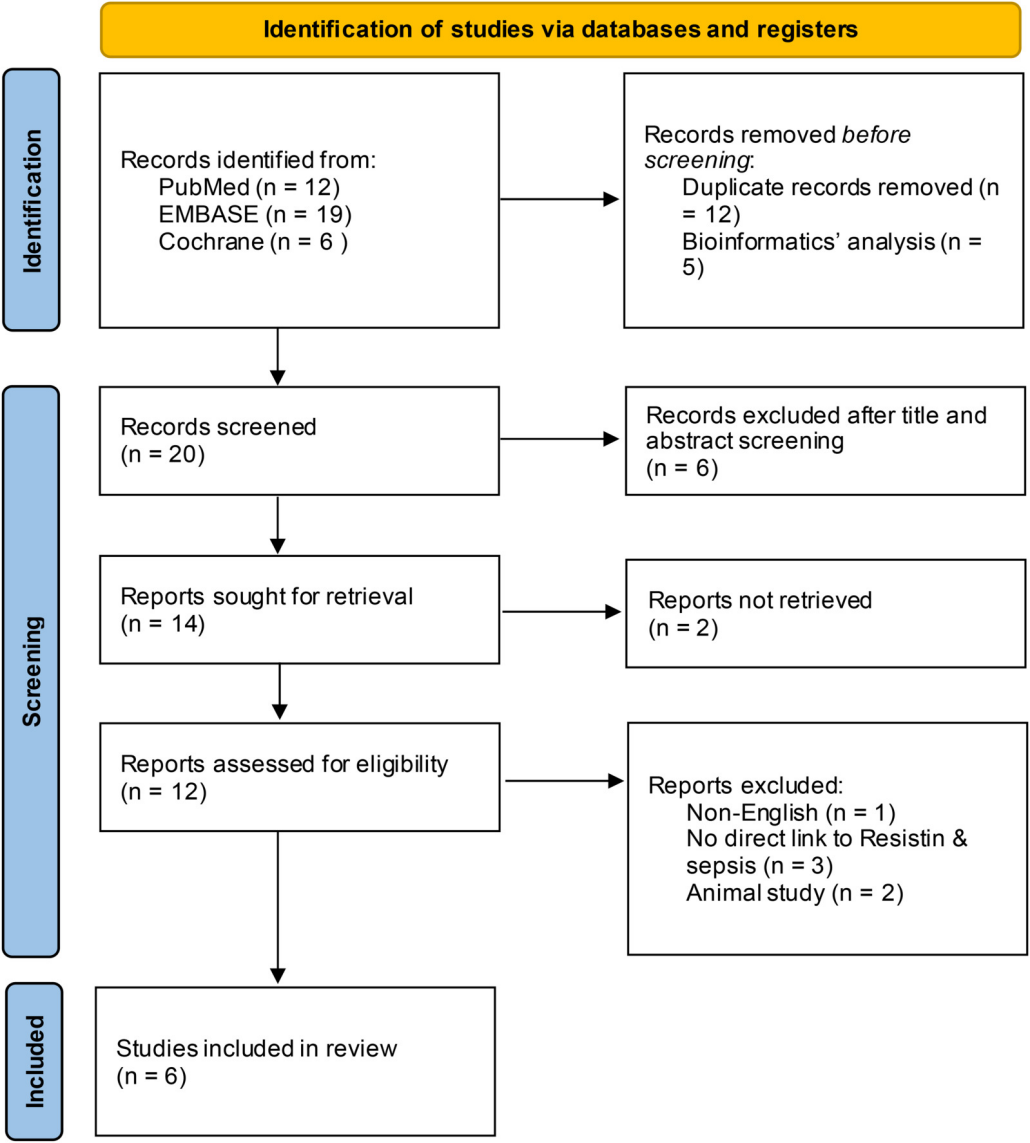
Flowchart of the article screening and selection process. After screening, 25 irrelevant articles were excluded, and six studies met the inclusion criteria after non-English language, animal and unrelated studies were removed.

This investigation identified 258 original articles through searches in the three medical databases, from which 205 duplicate articles and bioinformatic analyses were excluded. Following title and abstract screening, 25 irrelevant articles were excluded. After eliminating non-English language publications, animal studies, and those lacking a direct link to RETN levels and sepsis, six studies ultimately met our inclusion criteria (13, 14, 16–19). Figure 1 illustrates the selection process. The detailed characteristics and data for each included study are presented in Table 1.

**Table 1:** Characteristics of the 6 studies included in this meta-analysis.

Reference	Region	Study design	Outcome	Sample size (Cases/Controls)	Age	Biomarker	Cut-off *	Measurement	Sensitivity (%)	Specificity (%)	AUC
Saboktakin et al., 2019 (16)	Iran	Prospective	Paediatric sepsis	36 (24/12)	Under 12 years of age	Resistin	5.2	ELISA	82.4	72	0.864
Khattab et al., 2018 (13)	Egypt	Prospective	Culture-proven LOS	90 (60/30)	Neonate (term or late preterm)	Resistin	21.5	ELISA	100	96.7	0.999
						CRP	5	immunoturbidometry	100	100	1
Cekmez et al., 2011 (18)	Turkey	Prospective	Culture-proven LOS	105 (62/43)	am	Resistin	8	ELISA	93	95	0.912
						CRP	0.82	immunoturbidometry	82	79	0.845
Aliefendioglu et al., 2014 (14)	Turkey	Prospective	Culture-proven EOS	86 (43/43)	Neonate (preterm infants)	Resistin	50	ELISA	73.7	45.8	0.74
Gokmen et al., 2013 (17)	Turkey	Prospective	Culture-proven EOS and LOS	58 (20/38)	Neonate (<32 weeks preterm infants)	Resistin	28.1	ELISA	90	90.1	0.94
						CRP	1	immunoturbidometry	50	97.7	0.827
Ozdemir and Elgormus, 2017 (19)	Turkey	Prospective	Culture-proven EOS	62 (29/33)	Neonate (term or near term)	Resistin	36.8	ELISA	76	67	0.72
						CRP	4.15	immunoturbidometry	83	61	0.84

* Resistin, ng/ml; CRP, mg/dl.

EOS, early-onset sepsis; LOS, late-onset sepsis.

Two reviewers independently extracted relevant information from all the articles concerning the key study design and characteristics of the study population, including the study name, year, design, region, assay method, testing time, cut-off values, sepsis onset, patient characteristics and numbers, and outcome data (TP, FP, FN, and TN). Any disagreements were resolved through a consensus or, if necessary, by consulting a third reviewer.

### 2.3 Quality assessment

**Figure 2:**
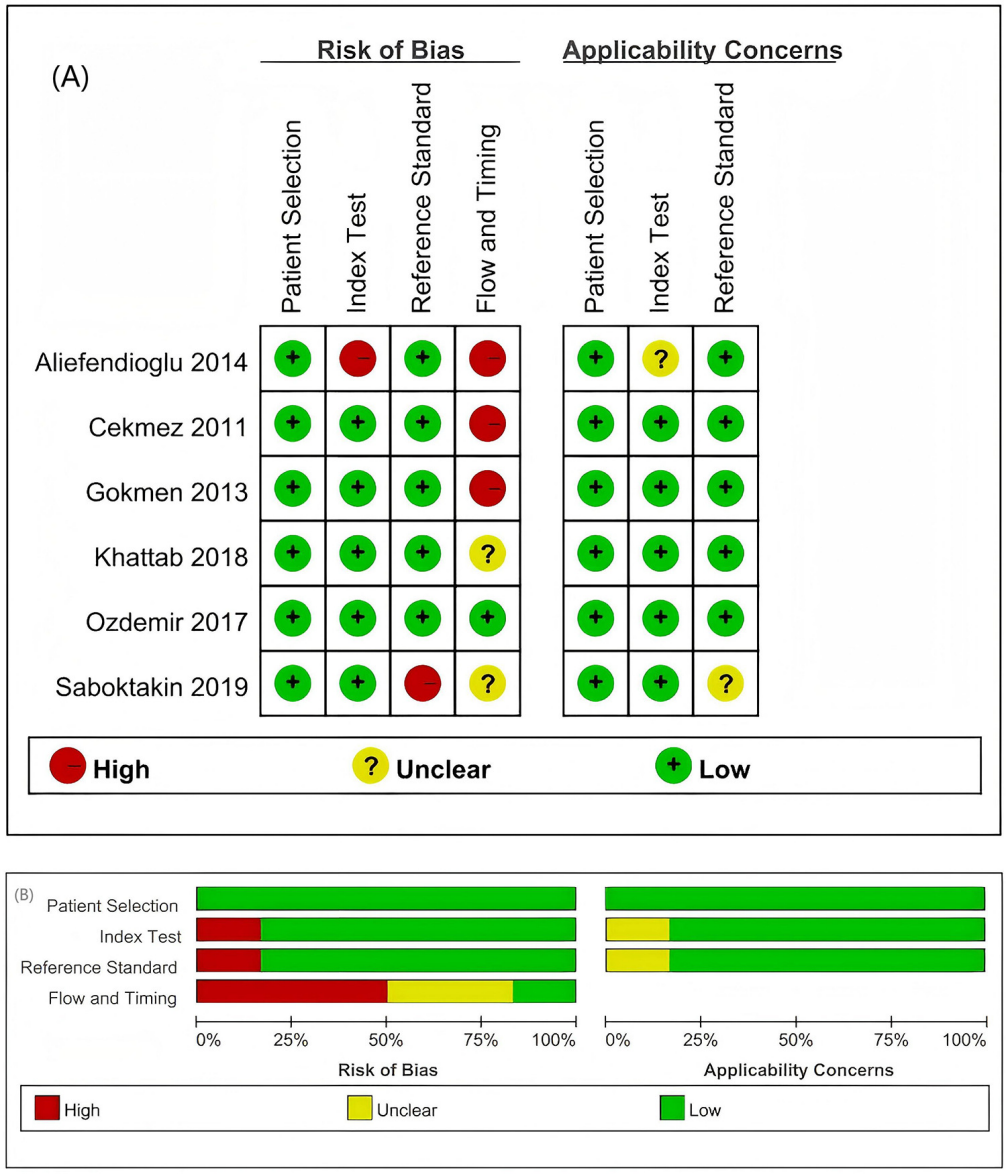
Summary of the risk of bias and applicability concerns based on the judgement of the review authors regarding each domain for each included study. **(A)** Risk of bias in the included studies. **(B)** Evaluation of the risk of bias in the included studies on patients with sepsis. Two studies exhibited an unclear risk (marked yellow) in the patient selection domain; one study demonstrated a high risk (red) in the reference standards, with another showing an unclear risk, whereas flow and timing assessments identified three studies with a high risk and one with an unclear risk.

The quality of the studies was evaluated independently by two reviewers using the QUADAS-2 tool, following the recommended methodologies outlined in the Cochrane Handbook for Diagnostic Test Accuracy Reviews, with each item rated as a low risk of bias, a high risk of bias, or an unclear bias (20). The results of the bias risk assessment conducted using the QUADAS-2 tool are illustrated in Figure 2. High-risk assessments are indicated by red circles, low-risk assessments are indicated by green circles, and unclear risk assessments are indicated by yellow circles. Uncertainties and unclear risks denote insufficient clarity and a lack of definitive judgement associated with the limited details of the studies.

In terms of patient selection, two studies were classified as having an unclear risk of bias regarding the consecutive or random sampling of the enrolled patients (14, 18). For the reference standard, the articles by Aliefendioglu et al. and Saboktakin et al. were assessed as having high and unclear risks of bias, respectively (14, 16). Regarding flow and timing, one study was deemed to have an unclear risk of bias, whereas three studies were classified as having a high risk of bias (13, 14, 17, 18).

### 2.4 Statistical analysis

The quality assessment results of the included studies were generated using the RevMan 5.4 software. Statistical analyses were conducted with the Meta-DiSc 1.4 and STATA 17.0 software. The overall diagnostic performances of RETN and CRP levels in diagnosing neonatal and paediatric sepsis were evaluated using the summary receiver operating characteristic (SROC) curves. Heterogeneity among the included studies was assessed using the Cochran Q statistic and quantified with the *I*^2^ statistic, which ranges from 0% to 100% (21). A *P* value of the *Q* test < 0.05 and an *I*^2^ index ≥50% indicated moderate heterogeneity, necessitating a discussion of its sources. Deek's funnel plot asymmetry test was used to assess publication bias in the included literature (22). If the result of the Deek's symmetry test yielded *P* < 0.05, the presence of publication bias was suggested.

## 3 Results

### 3.1 Diagnostic accuracy

**Figure 3:**
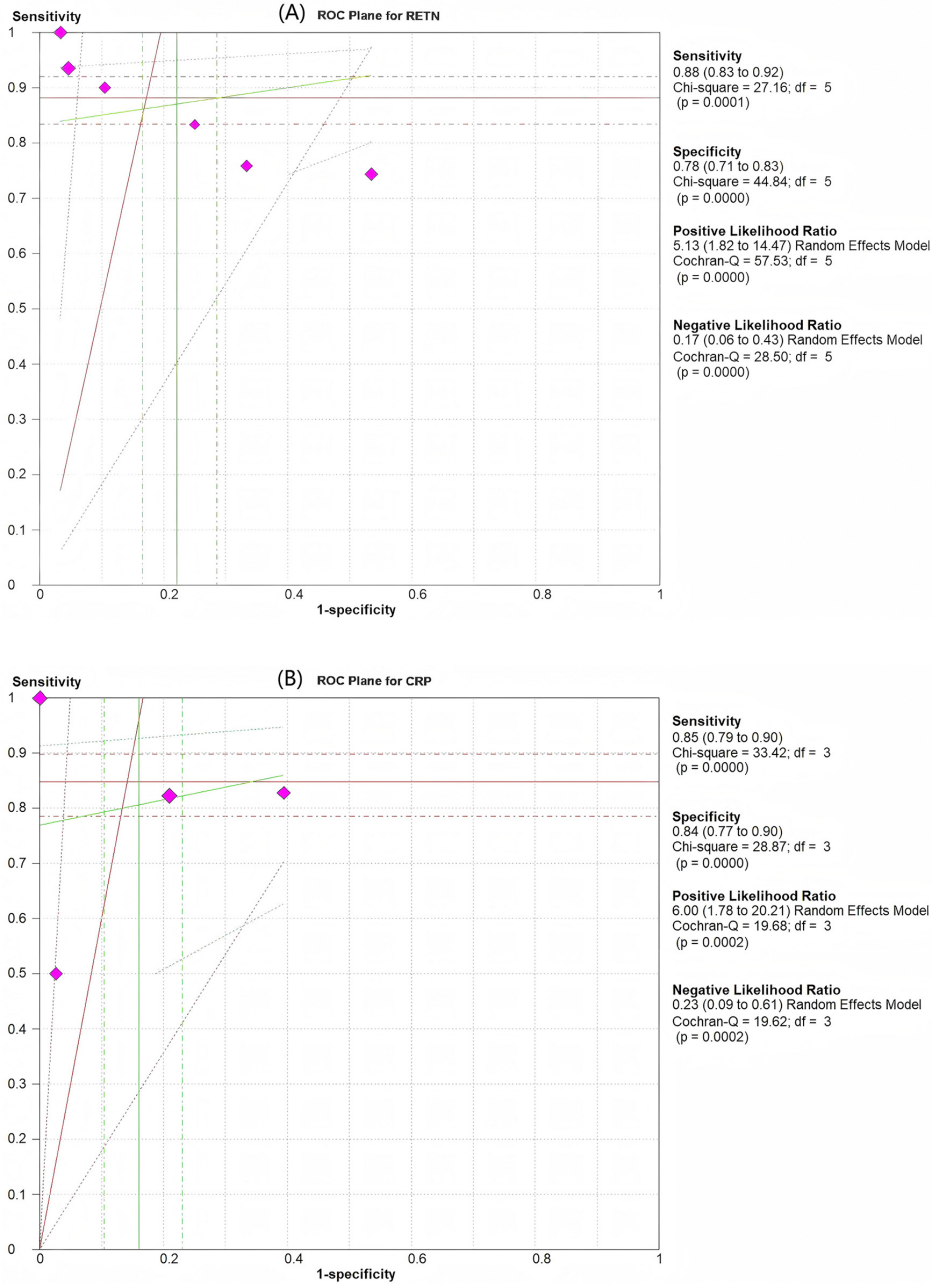
Pooled results of the studies assessing the diagnostic accuracy of resistin and CRP levels for the prediction of sepsis in infants and children. **(A)** ROC plane for resistin. **(B)** ROC plane for CRP. The diamond-shaped symbol marked in purplish red represents one study.

**Figure 4:**
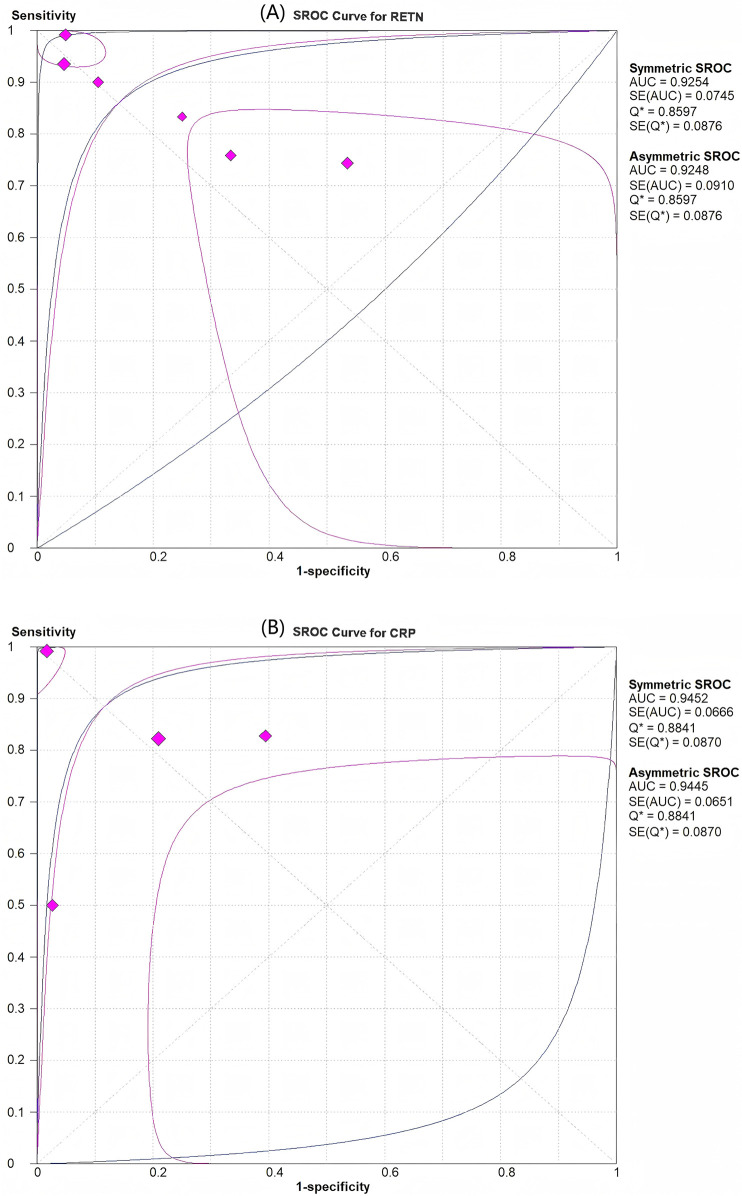
SROC curves for assessment of the diagnostic accuracy of resistin and CRP levels for predicting sepsis in infants and children. **(A)** Resistin had an area under the curve (AUC) of 0.925. **(B)** CRP had an AUC of 0.945.

Among the six articles that met our inclusion criteria, we examined the correlation between RETN levels and neonatal and paediatric sepsis. The meta-analysis results revealed that RETN testing had a greater sensitivity than specificity, whereas CRP testing had a greater specificity than sensitivity for diagnosing sepsis in neonates and children. The pooled sensitivity and specificity estimates for RETN levels were 0.88 (95% CI, 0.83–0.92) and 0.78 (95% CI, 0.71–0.83), respectively (Figure 3A). The positive likelihood ratio (LR+) of 5.13 (95% CI, 1.82–14.47) for the RETN test was sufficiently elevated to be used as a rule-in test, whereas the high negative likelihood ratio (LR−) of 0.17 (95% CI, 0.06–0.43) was inadequate to lower the pretest probability to a level that would allow for the safe exclusion of sepsis (Figure 3A). We constructed SROC curves for both RETN and CRP. The area under the curve (AUC) for RETN was 0.925 ± 0.074 (Figure 4A).

CRP had an AUC of 0.945 ± 0.067 (Figure 4B). The pooled sensitivity and specificity estimates for CRP were 0.85 (95% CI, 0.79–0.90) and 0.84 (95% CI, 0.77–0.90), respectively (Figure 3B). The LR+ of 6.00 (95% CI, 1.78–20.21) for the CRP test was sufficiently high to serve as a rule-in test, whereas the elevated LR− at 0.23 (95% CI, 0.09–0.61) could not diminish the pretest probability to a level that would allow for the safe exclusion of sepsis.

The diagnostic odds ratio (OR) for RETN was 36.20 (95% CI, 6.16–212.68), whereas that for CRP was 33.34 (95% CI, 6.16–180.43), as illustrated in Figure 3.

### 3.2 Heterogeneity assessment

The heterogeneity observed in sensitivity (*I*^2^ = 81.6%), specificity (*I*^2^ = 88.8%), positive LR (*I*^2^ = 91.3%), negative LR (*I*^2^ = 82.5%), and diagnostic odds ratio (*I*^2^ = 87.8%) was substantial according to forest plot results (see Supplementary Figures). There was notable variability in the ages of the patients (from newborns to children) and a broad range of cut-off values (5.2–50.0 ng/ml). A subgroup analysis was conducted on the basis of the timing of sepsis [early-onset sepsis [EOS] vs. late-onset sepsis [LOS]], population (term or near-term infants vs. others), geographical location (Turkey), and sample size (≤80 vs. >80) to explore the heterogeneity in diagnostic accuracy (*I*^2^ sensitivity) (Table 2).

**Table 2:** Subgroup analysis for assessing the diagnostic accuracy of resistin levels in predicting sepsis in infants and children.

Subgroup	No. of studies	Pooled sensitivity (%) (95% CI)	Pooled specificity (%) (95% CI)	Pooled LR+ (95% CI)*	Pooled LR− (95% CI)^†^	Pooled DOR 95% CI	*I*^2^ sensitivity %
Sample size (≥80)	3	91 (85–95)	78 (69–85)	7.99 (0.31–204.91)	0.09 (0.01–0.91)	101.44 (1.38–7,432)	90.9
Sample size (<80)	3	82 (71–90)	78 (68–87)	3.78 (1.67–8.54)	0.25 (0.13–0.47)	17 (4.02–71.80)	0
Population (term or near term)	3	93 (87–96)	87 (79–93)	9.2 (0.98–86.65)	0.08 (0.01–0.57)	131.8 (3.97–4,374)	88.3
Population (others)	3	80 (71–88)	68 (57–77)	3.24 (0.92–11.39)	0.27 (0.10–0.71)	12.78 (1.61–101.59)	17.2
Region (Turkey)	4	84 (78–90)	75 (67–81)	4.28 (1.34–13.67)	0.21 (0.08–0.59)	21.87 (2.83–169.26)	68.9
Timing of sepsis (EOS)	3	78 (68–86)	67 (57–75)	2.72 (1.13–6.56)	0.34 (0.16–0.73)	9.06 (1.71–48.05)	16

* LR+, positive likelihood ratio.

^†^ LR−, negative likelihood ratio.

EOS, early-onset sepsis.

The pooled sensitivity was greater in studies with 80 or more participants than in those with fewer than 80 participants, at 91% (95% CI, 85–95) vs. 82% (95% CI, 71–90), respectively. However, the specificity remained consistent at 78% (95% CI, 69–85) for both groups, with diagnostic odds ratios of 101.44 (95% CI, 1.38–7,432) and 17 (95% CI, 4.02–71.80), respectively. The pooled sensitivity was also greater in studies involving a population of term or near-term infants than in those involving other populations, at 93% (95% CI, 87–96) vs. 80% (95% CI, 71–88), whereas the specificity was significantly greater at 87% (95% CI, 79–93) vs. 68% (95% CI, 57–77), with diagnostic odds ratios of 131.8 (95% CI, 3.97–4,374) and 12.78 (95% CI, 1.61–101.59), respectively. The pooled sensitivity and specificity for the timing of sepsis (EOS) were 78% (95% CI, 68–86) and 67% (95% CI, 57–75), respectively, with a diagnostic odds ratio of 9.06 (95% CI, 1.71–48.05) and an *I*^2^ of 16%, respectively (Table 2).

### 3.3 Publication bias

**Figure 5:**
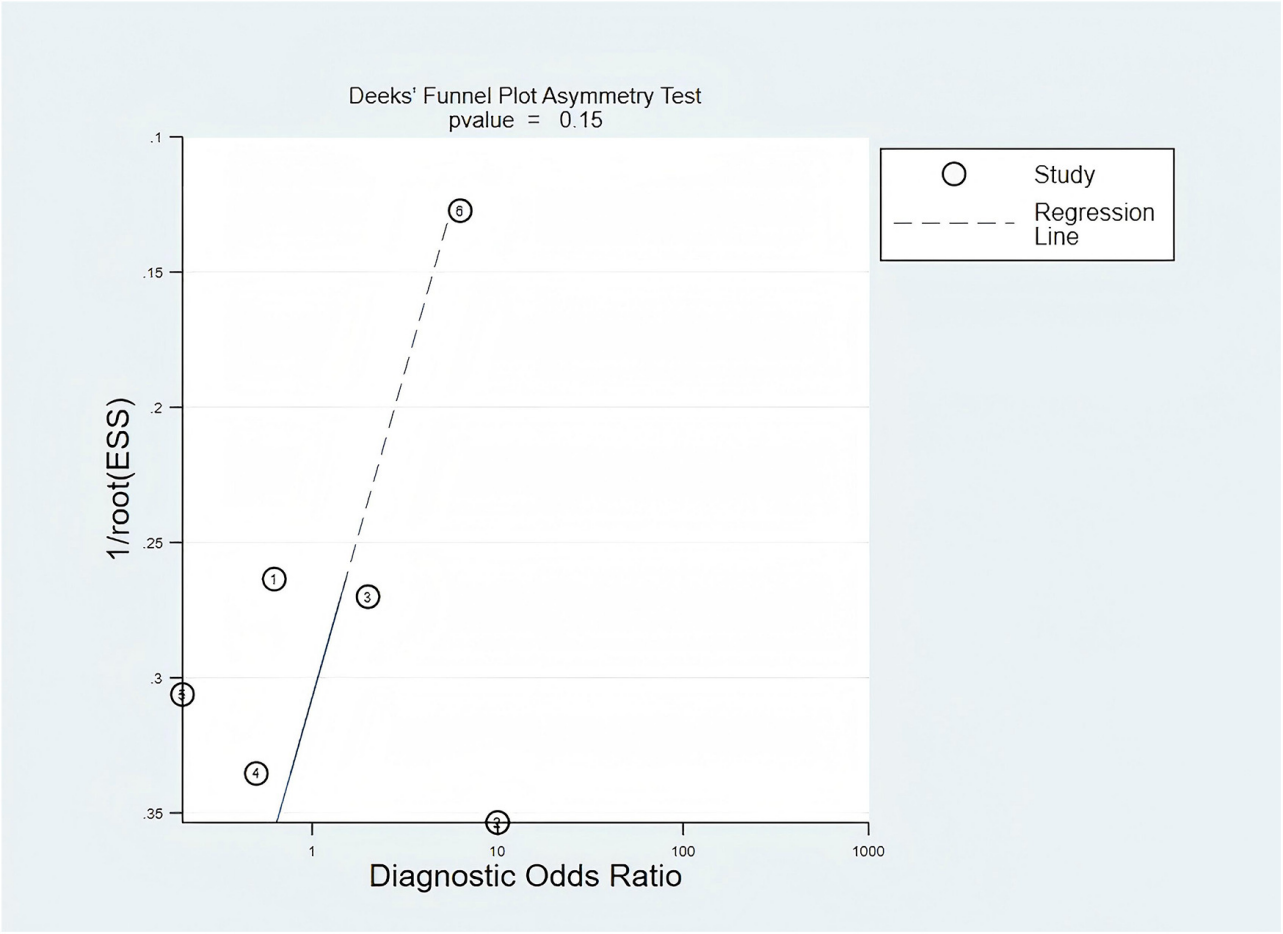
Publication bias was assessed using Deek's funnel plot. The value of *P* = 0.15 indicated that there was no significant publication bias among the included studies.

In the evaluation of publication bias, the results of the regression line test indicated that there was no publication bias (bias = −9.55; 95% CI, −24.31–5.22; *P* = 0.15). The findings from the Deek's funnel plot are illustrated in Figure 5.

## 4 Discussion

This meta-analysis demonstrated that the diagnostic accuracy of RETN levels for sepsis in neonates and children was not inferior to that of CRP levels. The SROC curve for RETN levels in the prediction of sepsis revealed a robust predictive ability. The overall pooled estimates for the sensitivity and specificity of elevated RETN concentrations in detecting sepsis were 0.88 (95% CI, 0.83–0.92) and 0.78 (95% CI, 0.71–0.83), respectively, with an AUC of 0.93. To our knowledge, this meta-analysis is the first to evaluate the relationship between RETN levels and sepsis and to compare the diagnostic accuracy of RETN levels with that of CRP levels.

RETN, identified as an adipokine in 2001, is minimally expressed in healthy individuals, but its level significantly increases upon activation of inflammatory mediators following infection or injury (5, 23). RETN promotes inflammatory cell activation, disrupts the immune balance, and damages vascular endothelial cells, thereby contributing to the pathogenesis of sepsis (24, 25). Research has indicated that RETN activates various cell types, leading to the production of proinflammatory cytokines, such as IL-6, IL-1β and tumour necrosis factor-alpha (TNF-α) through the Toll-like receptor 4 (TLR4)- or cyclase-associated protein 1 (CAP1)-mediated signalling pathways (26–29). The presence of RETN may also lead to an aberrant immune response in specific contexts and diseases, suggesting its role as a bidirectional immunomodulatory molecule (26). Additionally, RETN can induce endoplasmic reticulum stress, inhibit insulin-stimulated endothelial nitric oxide production, impair insulin signalling in vascular endothelial cells, and increase the production of reactive oxygen species, along with the increase of the mRNA expression of proinflammatory cytokines, ultimately resulting in endothelial cell dysfunction (30). However, RETN has been shown to increase autophagy in bovine alveolar macrophages by activating the adenosine monophosphate-activated protein kinase (AMPK) and mammalian target of rapamycin (mTOR) signalling pathways, potentially alleviating lipopolysaccharide (LPS)-induced inflammation (31). Consequently, the precise clinical significance of this molecule remains uncertain.

Several studies have shown that the RETN level may be a specific marker for the early identification of patients at increased risk of sepsis (32, 33). A study by Aliefendioglu established a RETN concentration cut-off of 50 ng/ml, yielding a sensitivity of 73.7% and a specificity of 45.8% for the early detection of neonatal sepsis (14). A study conducted by Saboktakin demonstrated that RETN levels could be used as indicators of sepsis in children admitted to the PICU, with a sensitivity of 0.824 and a specificity of 0.72 on the first day (16). And the diagnostic value of RETN was found to be limited compared with that of other inflammatory markers, including C-reactive protein, procalcitonin, and IL-6 (13, 14, 16). However, Lan et al. reported that the specificity of the RETN level as a diagnostic marker for sepsis was 91.7%, indicating high accuracy (31). Cekmez et al. reported a notable diagnostic accuracy for RETN levels, with 93% sensitivity and 95% specificity for neonatal sepsis (18). Our findings support the RETN level as a reliable sepsis marker in infants and children.

Typically, there exists a trade-off between sensitivity and specificity in diagnostic accuracy tests, with an increase in sensitivity (true positive rate) often accompanied by a decrease in specificity (true negative rate). Therefore, relying solely on sensitivity and specificity may not provide the most accurate estimation of diagnostic accuracy. Alternatively, the area under the SROC curve, or AUC, may serve as a more reliable metric. The AUC ranges from 0.50 to 1.00 and correlates with overall diagnostic accuracy. The current study revealed that the AUC for the SROC curve of RETN was 0.93, indicating that the RETN level is a valuable diagnostic marker for sepsis in neonates and children.

Given the high mortality and morbidity associated with neonatal and paediatric sepsis, diagnostic tests with high sensitivity and a high negative predictive value are highly important. CRP, a well-established biomarker for the early diagnosis of sepsis, has extensively been used in clinical settings (34–36). Our meta-analysis revealed that the sensitivity of RETN surpassed that of CRP, although its specificity was lower. Our study revealed high AUCs for both RETN and CRP, indicating that these two biomarkers exhibit enhanced diagnostic accuracy for sepsis. Compared with CRP levels, RETN levels were found to be equally effective in diagnosing sepsis in neonates and children. Furthermore, RETN levels provide critical diagnostic gains by improving early sepsis detection when traditional markers remain equivocal. The 3% higher pooled sensitivity of RETN than that of CRP (88% vs. 85%) in our meta-analyses enhances the rule-out capability, potentially reducing the number of missed cases during the initial triage. The biomarker's rapid elevation within 2–4 h of infection offers a temporal advantage over CRP (typically rising after 6–8 h), enabling earlier antibiotic decisions in time-sensitive scenarios like paediatric and neonatal sepsis (37).

The cut-off values for RETN levels in the diagnosis of sepsis have not been consistently reported across studies, even when the same assay is used, which significantly impedes the clinical application of this biomarker. Macdonald et al. reported that sustained elevations of RETN levels were linked to severe sepsis or septic shock, with the levels ranging from 36.5 to 50.8 ng/ml within 30 h after sepsis onset in adults (38). In our investigation, the reported cut-off values varied from 5.2 to 50 ng/ml for RETN and from 0.82 to 5 mg/ml for CRP. The variability in cut-off values may be attributed to differences in sepsis severity, study designs, clinical environments, and sample types (33). Therefore, future investigations should aim to eliminate the influence of these confounding factors on RETN levels to establish clinically relevant cut-off values.

This meta-analysis has several limitations. First, some results exhibited high heterogeneity. While we identified certain sources of heterogeneity through various methods, some remain unclear. Second, only six studies that assessed the diagnostic value of RETN were included because of the limited data availability. Finally, most of the studies were conducted on European populations, and the findings may not be generalizable to other ethnic groups (13, 14, 17–19). These factors may introduce a risk of bias in the results of the current study.

In conclusion, on the basis of the currently available evidence, RETN levels are highly valuable for early detection of neonatal and paediatric sepsis. Further prospective controlled studies with adequate sample sizes that encompass all predisposing factors for sepsis are necessary to elucidate the relationship between RETN levels and sepsis.

## Data Availability

The original contributions presented in the study are included in the article/Supplementary Material, further inquiries can be directed to the corresponding author.
