# Supplementary material for: Comparison of the diagnostic accuracy of resistin and CRP levels for sepsis in neonates and children: a systematic review and meta-analysis
Source: Front Pediatr. 2025 May 9;13:1555671. doi: 10.3389/fped.2025.1555671 (PMC12098288; doi:10.3389/fped.2025.1555671)

Supplement 1. The search strategy in pubmed.

#1 ((("Resistin"[Mesh]) OR Resistin [Title/Abstract]) OR RENT [Title/Abstract])

#2 ((((("Pediatric Sepsis"[Mesh]) OR Pediatric Sepsis[Title/Abstract]) OR Sepsis, Pediatric [Title/Abstract]) OR Sepsis, Child[Title/Abstract]) OR Sepsis, Children[Title/Abstract])

#3 ((((((((((((((((("Neonatal Sepsis"[Mesh]) OR Neonatal Sepsis[Title/Abstract]) OR Sepsis, Neonatal[Title/Abstract]) OR Sepsis, Neonatal[Title/Abstract]) OR Neonatal Late-Onset Sepsis[Title/Abstract]) OR Late-Onset Sepsis, Neonatal[Title/Abstract]) OR Late-Onset Sepsis, Neonatal[Title/Abstract]) OR Neonatal Late Onset Sepsis[Title/Abstract]) OR Neonatal Late-Onset Sepsis[Title/Abstract]) OR Sepsis, Neonatal Late-Onset[Title/Abstract]) OR Sepsis, Neonatal Late-Onset[Title/Abstract]) OR Neonatal Early-Onset Sepsis[Title/Abstract]) OR Early-Onset Sepsis, Neonatal[Title/Abstract]) OR Early-Onset Sepsis, Neonatal[Title/Abstract]) OR Neonatal Early Onset Sepsis[Title/Abstract]) OR Neonatal Early-Onset Sepsis[Title/Abstract]) OR Sepsis, Neonatal Early-Onset[Title/Abstract]) OR Sepsis, Neonatal Early-Onset[Title/Abstract]

#4 # 2 OR #3

#5 #1 AND #4


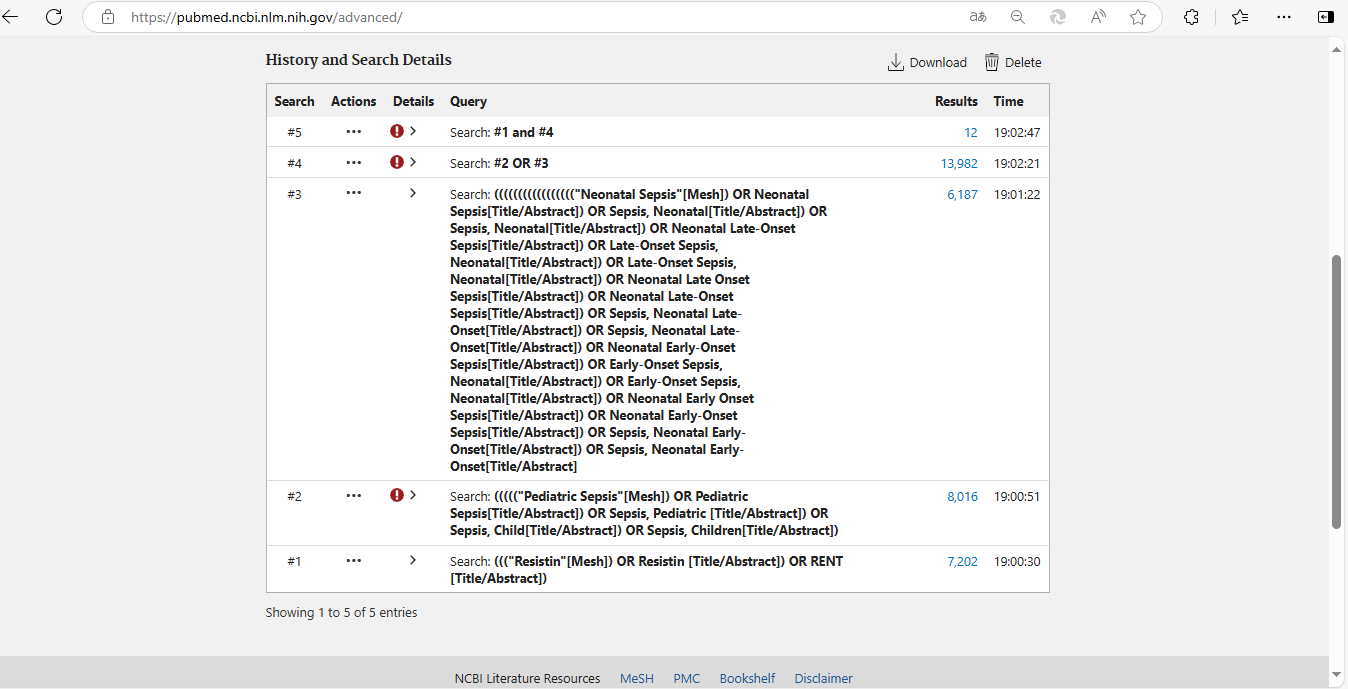


Supplement 2. The search strategy in the Cochrane library.

#1 ((Resistin) OR RENT)

#2 (Pediatric Sepsis) OR (Sepsis, Pediatric) OR (Sepsis, Child) OR (Sepsis, Children)

#3 (Neonatal Sepsis) OR (Sepsis, Neonatal) OR (Sepsis, Neonate) OR (Neonatal Late-Onset Sepsis) OR (Late-Onset Sepsis, Neonatal) OR (Late-Onset Sepsis, Neonate) OR (Neonatal Early-Onset Sepsis) OR (Early-Onset Sepsis, Neonatal) OR (Early-Onset Sepsis, Neonate) OR (Neonatal Early Onset Sepsis) OR (Sepsis, Neonatal Early-Onset)

#4 # 2 OR #3

#5 #1 AND #4


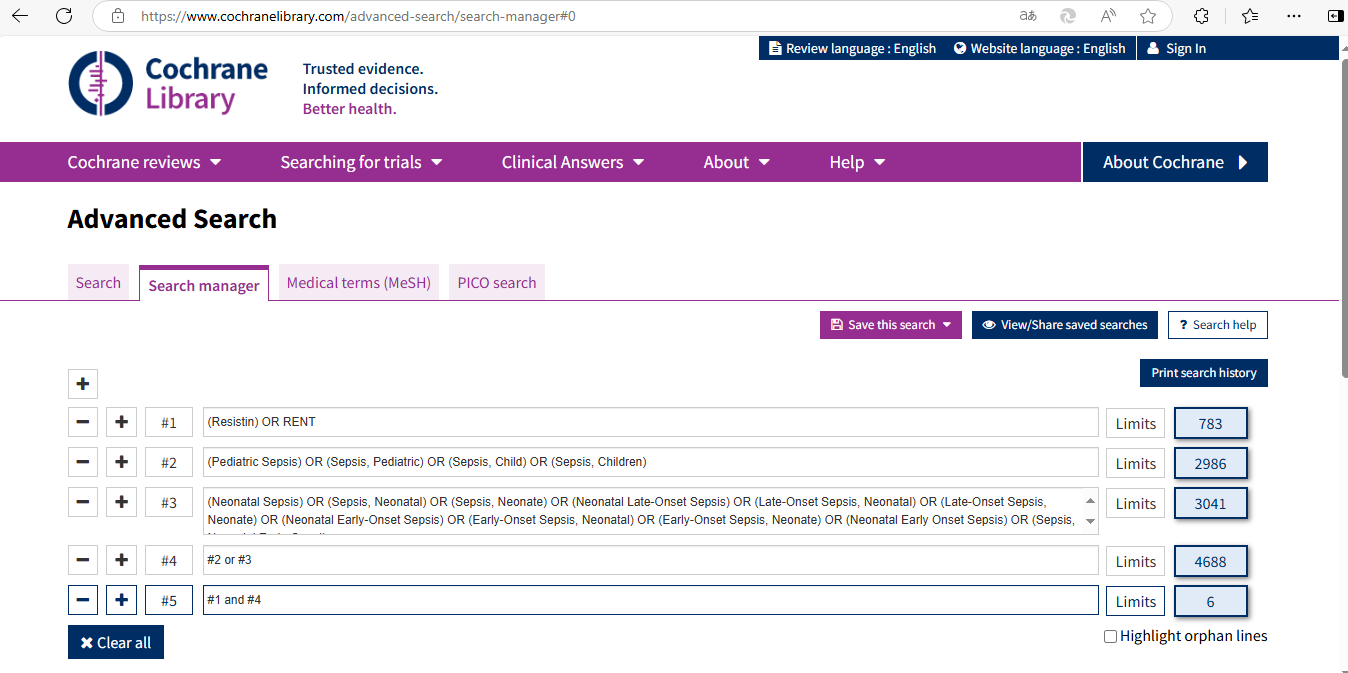


Supplement 3. The search strategy in Embase.

((Resistin) OR RENT)) and ((Pediatric Sepsis) OR (Sepsis, Pediatric) OR (Sepsis, Child) OR (Sepsis, Children)) and ((Neonatal Sepsis) OR (Sepsis, Neonatal) OR (Sepsis, Neonate) OR (Neonatal Late-Onset Sepsis) OR (Late-Onset Sepsis, Neonatal) OR (Late-Onset Sepsis, Neonate) OR (Neonatal Early-Onset Sepsis) OR (Early-Onset Sepsis, Neonatal) OR (Early-Onset Sepsis, Neonate) OR (Neonatal Early Onset Sepsis) OR (Sepsis, Neonatal Early-Onset))/br


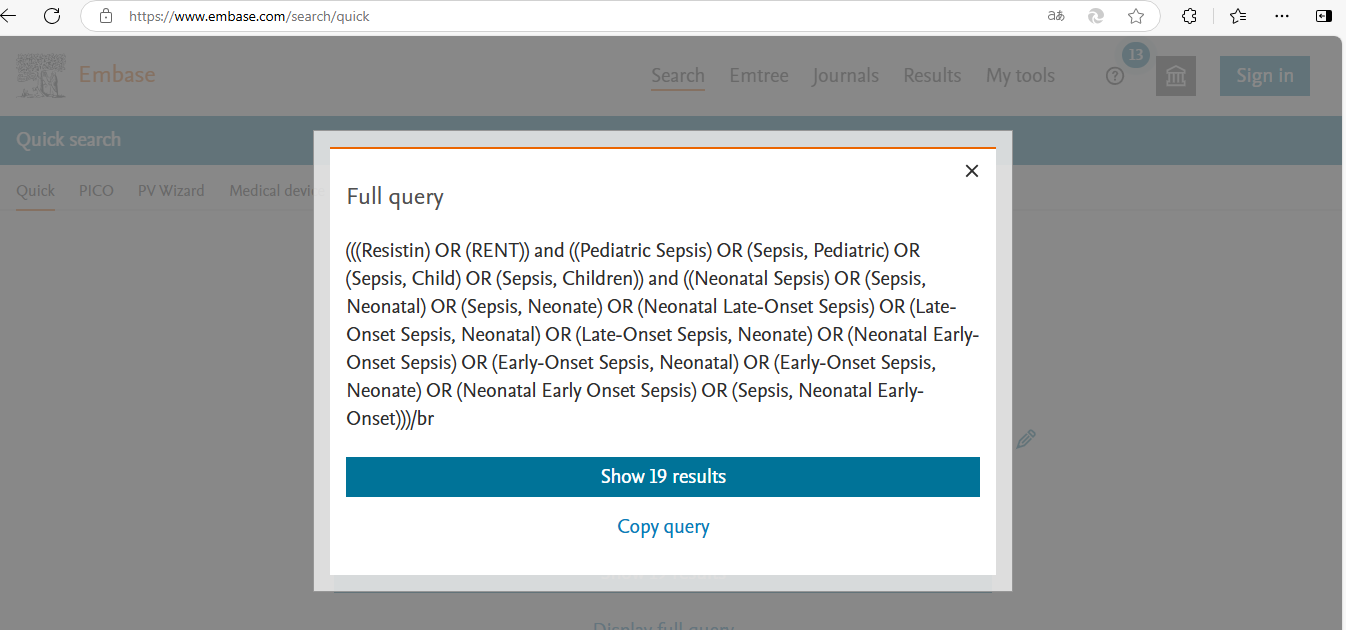

Supplement: Supplementary file 1 [file Datasheet1.docx]
