# Supplementary figures and images for: Comparison of the diagnostic accuracy of resistin and CRP levels for sepsis in neonates and children: a systematic review and meta-analysis
Source: Front Pediatr. 2025 May 9;13:1555671. doi: 10.3389/fped.2025.1555671 (PMC12098288; doi:10.3389/fped.2025.1555671)

## Slide 1
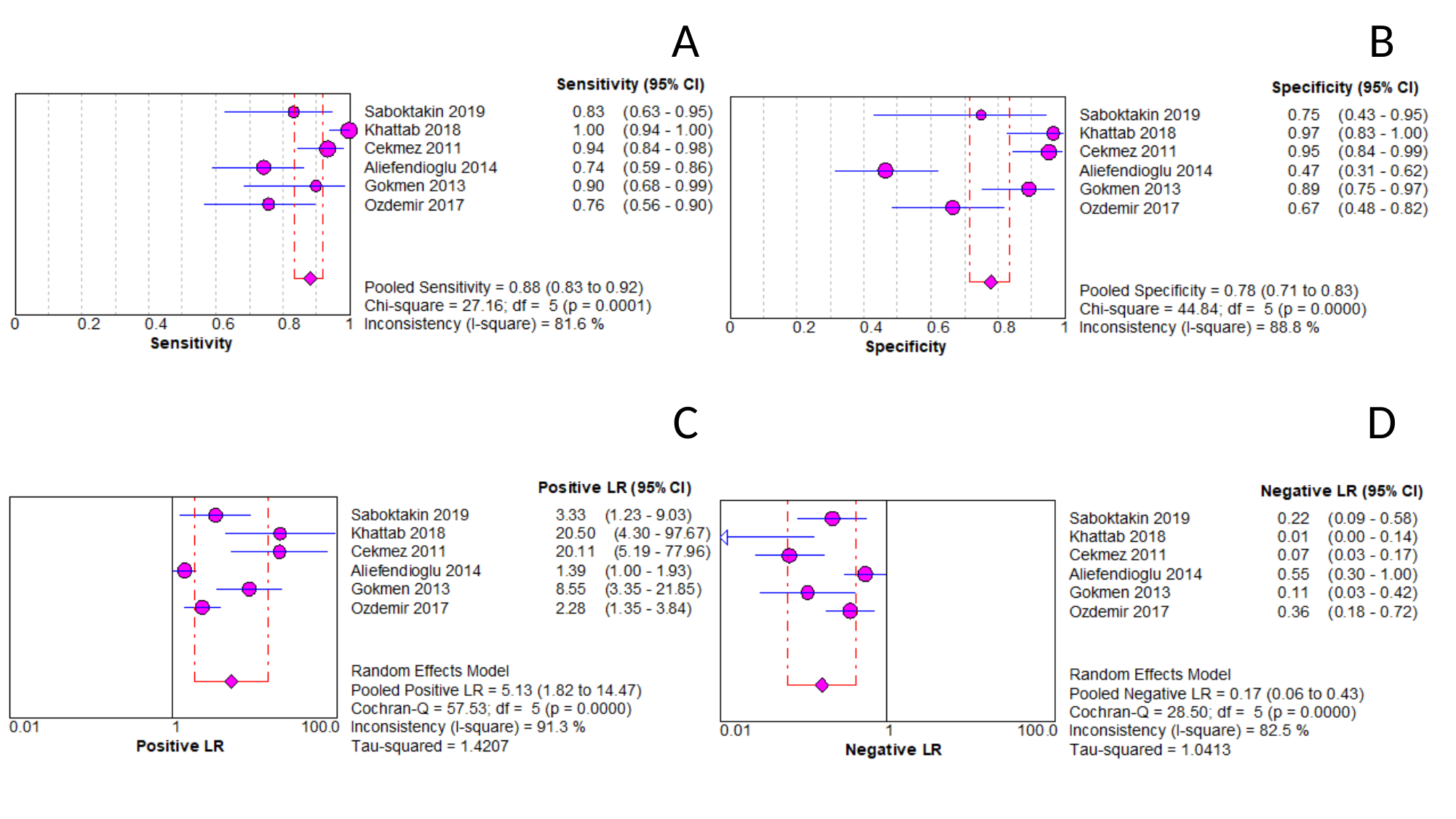

A
B
D
C

## Slide 2
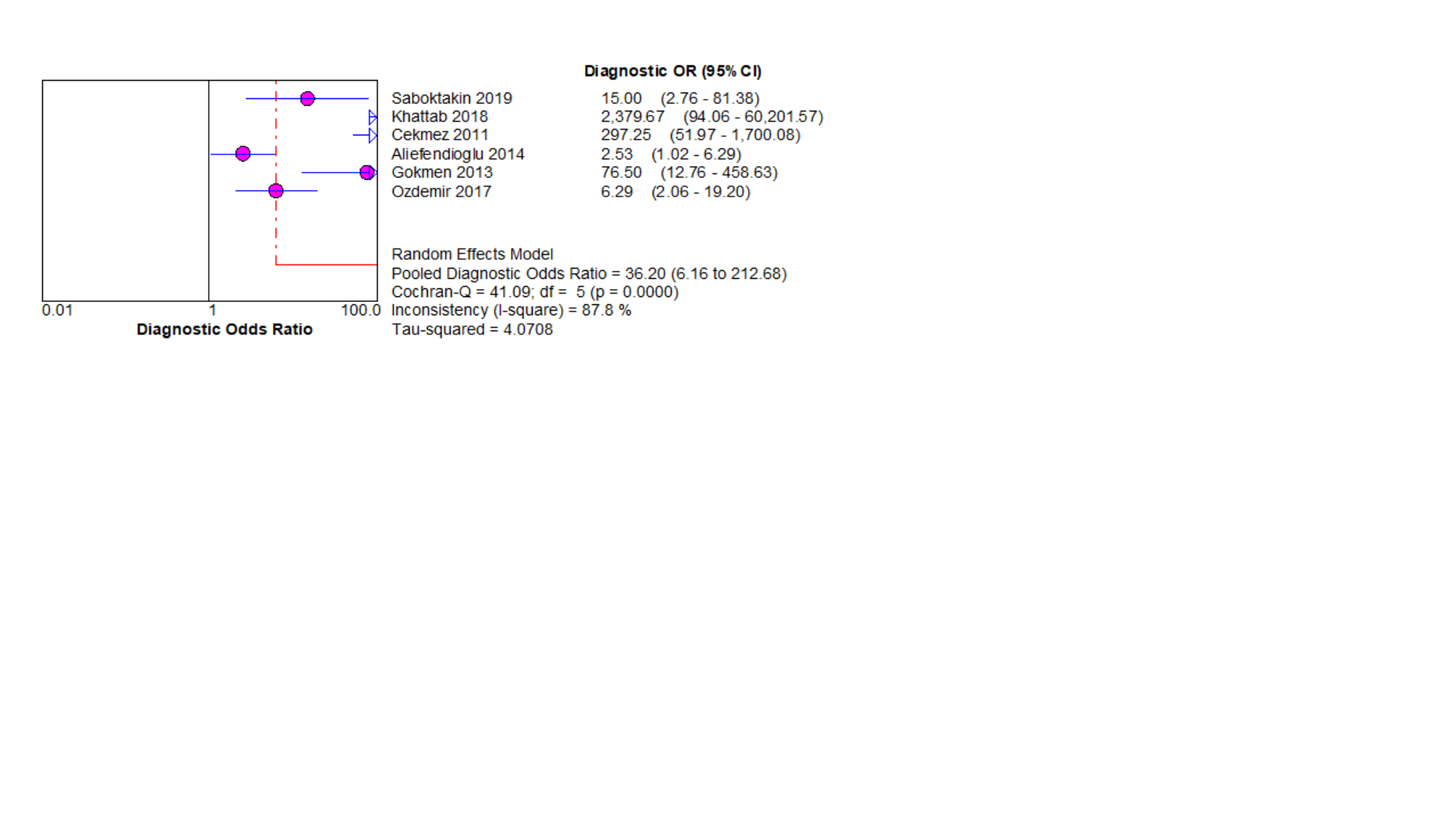

Supplement: Supplementary file 2 [file Presentation1.pptx]
